# Development of a Novel Prediction Model for Red Blood Cell Transfusion Risk in Cardiac Surgery

**DOI:** 10.3390/jcm12165345

**Published:** 2023-08-17

**Authors:** Ordoño Alonso-Tuñón, Manuel Bertomeu-Cornejo, Isabel Castillo-Cantero, José Miguel Borrego-Domínguez, Emilio García-Cabrera, Luis Bejar-Prado, Angel Vilches-Arenas

**Affiliations:** 1 Department of Anesthesia and Reanimation, Virgen del Rocio University Hospital, 41013 Seville, Spain; ordaft@hotmail.com (O.A.-T.);; 2 Department of Obstetric and Gynecology, Maternity and Children Hospital, Virgen del Rocio University Hospital, 41013 Seville, Spain; 3 Department of Cardiovascular Surgery, Virgen del Rocio University Hospital, 41013 Seville, Spain; jmborregod@gmail.com; 4 Department of Preventive Medicine and Public Health, University of Seville, 41004 Seville, Spain; lmbprado@us.es (L.B.-P.);; 5 Department of Preventive Medicine and Public Health, Virgen Macarena University Hospital, 41009 Seville, Spain

**Keywords:** cardiac surgery, transfusion risk, score development

## Abstract

Background: Cardiac surgery is a complex and invasive procedure that often requires blood transfusions to replace the blood lost during surgery. Blood products are a scarce and expensive resource. Therefore, it is essential to develop a standardized approach to determine the need for blood transfusions in cardiac surgery. The main objective of our study is to develop a simple prediction model for determining the risk of red blood cell transfusion in cardiac surgery. Methods: Retrospective cohorts of adult patients who underwent cardiac surgery between 2017 and 2019 were studied to identify hypothetical predictors of blood transfusion. Finally, a multivariable logistic regression model was developed to predict the risk of transfusion in cardiac surgery using the AUC and the Hosmer–Lemeshow goodness-of-fit test. Results: We included 1234 patients who underwent cardiac surgery. Of the entire cohort, 875 patients underwent a cardiac procedure 69.4% [CI 95% (66.8%; 72.0%)]; 119 patients 9.6% [CI 95% (8.1%; 11.4%)] underwent a combined procedure, and 258 patients 20.9% [CI 95% (18.7; 23.2)] underwent other cardiac procedures. The median perioperative hemoglobin was 13.0 mg/dL IQR (11.7; 14.2). The factors associated with the risk of transfusion were age > 60 years OR 1.37 CI 95% (1.02; 1.83); sex female OR 1.67 CI 95% (1.24; 2.24); BMI > 30 OR 1.46 (1.10; 1.93); perioperative hemoglobin < 14 OR 2.11 to 51.41 and combined surgery OR 3.97 CI 95% (2.19; 7.17). The final model shows an AUC of 80.9% for the transfusion risk prediction [IC 95% (78.5–83.3%)]; *p* < 0.001]. Conclusions: We have developed a model with good discriminatory ability, which is more parsimonious and efficient than other models.

## 1. Introduction

Cardiac surgery, along with orthopedic surgery, consumes a significant proportion of global blood resources; this is one of the reasons why blood conservation strategies in cardiac surgery are highly adjusted [1]. Recent estimates suggest that 20–60% of patients undergoing cardiac surgery in the United States require perioperative transfusions. This means that more than 3 million patients receive more than 11 million transfused blood units [2]. Approximately 10% of all patients who undergo cardiac surgery suffer severe bleeding, and around 5% of patients who undergo this type of surgery will require urgent revision to correct bleeding and stabilize hemostasis [1].

In addition to high demand, blood transfusions in cardiac surgery are associated with an increase in postoperative morbidity and mortality [3]. A higher risk of infection [4], renal failure, cardiac events, neurological injuries, acute lung injuries related to transfusions, and circulatory overload are associated with transfusions [5] and the inherent risk of transfusion-transmitted infections [6].

Effective management of blood products is a key component of high-quality care in cardiac surgery [7]. Patient blood management (PBM) programs are becoming increasingly important for ensuring the proper management of blood products and for reducing the complications they produce [8], allowing for the standardization of practices and the reduction in blood transfusions [9]. One of the first steps to take is the preoperative evaluation of the risk of perioperative transfusion, as recommended by the American Society of Anesthesiologists and the European Society of Anesthesiology [1].

Numerous research studies have developed practical scoring systems to predict the likelihood of needing blood transfusions during cardiac surgery. These models include the TRUST model [10] and TRACK [11]. The TRUST model consists of eight predictors [10], while the subsequent TRACK system adopts a more concise approach with only five predictors while still demonstrating comparable predictive capacity [11].

However, when these models were externally validated in different cohorts, especially in regions such as Latin America and Asia [12,13], discrepant results were observed. Consequently, the ACTA-PORT [8] scoring system was developed. This model encompasses seven predictors, one of which is the EuroSCORE, a mortality risk score for cardiac surgery comprising 14 variables, which makes it difficult to calculate [14]. Furthermore, the ACTA-PORT model has been validated in patients of various ages [15] but lacks external validation outside the United Kingdom. Due to these variations in predictive performance and validation, there is still considerable divergence in the management of blood products during cardiac surgery between different institutions and practitioners [16]. Taking into account these challenges, there is a need for simpler alternative approaches to developing risk stratification models for blood transfusions utilizing variables that can be easily applied in any medical center.

The main objective of our study is to develop a parsimonious prediction model for the risk of red blood cell transfusion in cardiac surgery, evaluate its prediction capacity, and compare it with current risk stratification score prediction.

## 2. Materials and Methods

This retrospective observational cohort study was conducted at Virgen del Rocio University Hospital in Seville, Spain, between 2017 and 2019 and was approved by the Biomedical Research Ethics Committee. The study included all planned cardiac surgeries (such as valvular or bypass, combined valve-coronary artery bypass grafting (CABG), valve–valve, closure of intra-atrial communication (IAC) and thoracic aortic surgery, among others) for patients 16 years or older during the study period. Surgeries such as cardiac transplantation and transaortic valve implantation (TAVI) were not included. All patients received follow-up during hospitalization after the cardiac procedure until discharge or death.

We included the following factors associated with risk transfusion:

- Sociodemographic and anthropometric characteristics: age, sex, weight, height, body mass index (BMI) and body surface area (BSA).
- Clinical and personal history: comorbidities and toxic habits.
- Cardiovascular risk factors: diabetes mellitus, arterial hypertension, dyslipidemia and EuroSCORE I. EuroSCORE II, which is the current risk metric for surgery-related mortality within 30 days, was not used in this study as our objective was to compare against the current transfusion risk model [8].
- Variables included in EuroSCORE I: chronic obstructive pulmonary disease (COPD), previous cardiac surgery, extracardiac arteriopathy, neurological dysfunction, serum creatinine >200 μmol/L, active infectious endocarditis, unstable angina, left ventricle ejection fraction (LVEF), recent myocardial infarction, pulmonary hypertension, surgery other than CABG, surgery on the thoracic aorta and post-infarct septal rupture.
- Variables related to the surgical intervention: time of extracorporeal circulation, complications. The surgical procedure was classified into three categories: one procedure, when patients underwent valvular surgery or CABG surgery; combined procedures when patients underwent a combined procedure like valve–valve surgery or valve–CABG surgery and other cardiac procedures, including other cardiothoracic procedures.
- Other variables related to transfusion control: need for transfusion of red blood cell concentrates, number of concentrates transfused, other blood products, pro-hemostatic and ACTA–PORT score.

A descriptive analysis was performed using qualitative variables, represented in tables as absolute frequencies and percentages. Quantitative variables were expressed using the median and standard deviation or interquartile range (IQR), depending on whether they followed a normal distribution. The confidence interval of proportion was calculated using the Wald method and the form median was calculated using the bootstrap method [17].

The primary outcome was defined as the transfusion of at least one unit of total blood or one packed red blood cell to the patient throughout the perioperative period. The univariate association between the hypothetical predictors of blood transfusion and the outcome variable was analyzed using logistic regression analysis. In this method, the best set of predictor variables was selected from the variables that were significantly related to the dependent variable in the univariate analysis at a significance level below 0.15. This univariate analysis was assessed using logistic regression. A total of 8 hypothetical variables that could be included in the final model were identified. The strategy for selecting the best regression equation was carried out in accordance with the Kleinbaum methodology [18]. The odds ratio was calculated for the variables included in the resulting model and their respective 95% confidence intervals, 95% CI.

The ability to discriminate between observed and predicted values was evaluated using the area under the curve (AUC). The goodness of fit of the models was evaluated using the Hosmer–Lemeshow goodness-of-fit test, calibration slope calculation, and calibration plots. The final power of the logistic regression model was evaluated with the pwrss package [19], statistical power = 0.925.

All statistical analyses were performed using IBM’s Statistical Package for the Social Sciences (SPSS) software (version 26.0; Chicago, IL, USA), and R version 4.0.3 [20].

## 3. Results

### 3.1. Characteristics of the Cohort

**Figure 1:**
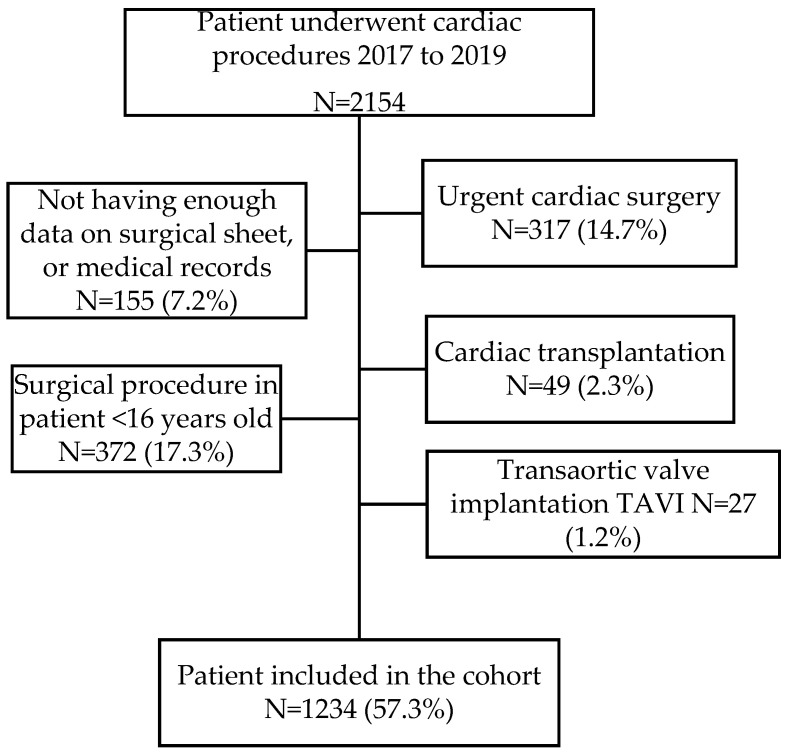
Flowchart of inclusion.

During the study period, a total of 2154 surgical procedures were performed. Of them, 920 (42.7%) were excluded because they did not meet the inclusion criteria (Figure 1), and 1234 adult patients undergoing cardiac surgery were included. The median age of the patients was 66 years, IQR (58; 73), and 63.9% were men [CI 95% (61.2; 66.6)]. The median BMI was 28, IQR (25.3; 31.8), and the BSA was 1.8, IQR (1.7; 2.0). The most common comorbidity was arterial hypertension (HBP), which affected 65.1% of the patients [CI 95% (62.4; 67.7)]. At the time of surgery, 18.4% of the patients [CI 95% (16.3; 20.6)] were reported as current smokers. Table 1 summarizes all the characteristics of the study cohort.

Of the entire cohort, 875 patients underwent a cardiac procedure (69.4% [CI 95% (66.8%; 72.0%)]); 119 patients underwent combined surgery (9.6% [CI 95% (8.1%; 11.4%)]), and 258 patients underwent other cardiac procedures (20.9% [CI 95% (18.7; 23.2)]). The median perioperative hemoglobin level was 13.0 mg/dL, IQR (11.7; 14.2). The median estimated mortality risk based on EuroSCORE I was 4.4% [CI 95% (2.4%; 7%)]. Table 2 shows the perioperative and operative characteristics.

### 3.2. Risk Factors Associated with Red Blood Cell Transfusion in Cardiac Surgery

During the perioperative period, 733 (59.4%) patients in the entire cohort received blood transfusions. The transfused group exhibited a median preoperative hemoglobin level of 12.2 g/dL, while the overall median was 13.0 g/dL. The combined procedure group had the highest transfusion demand, with 86.6% of the 119 surgeries analyzed requiring at least one transfusion. Patients who received transfusions had a longer median stay in the ICU and in the hospital. Table 3 describes all the factors associated with the need for transfusions.

### 3.3. Prediction Model of the Risk of Red Blood Cell Transfusion in Cardiac Surgery

**Figure 2:**
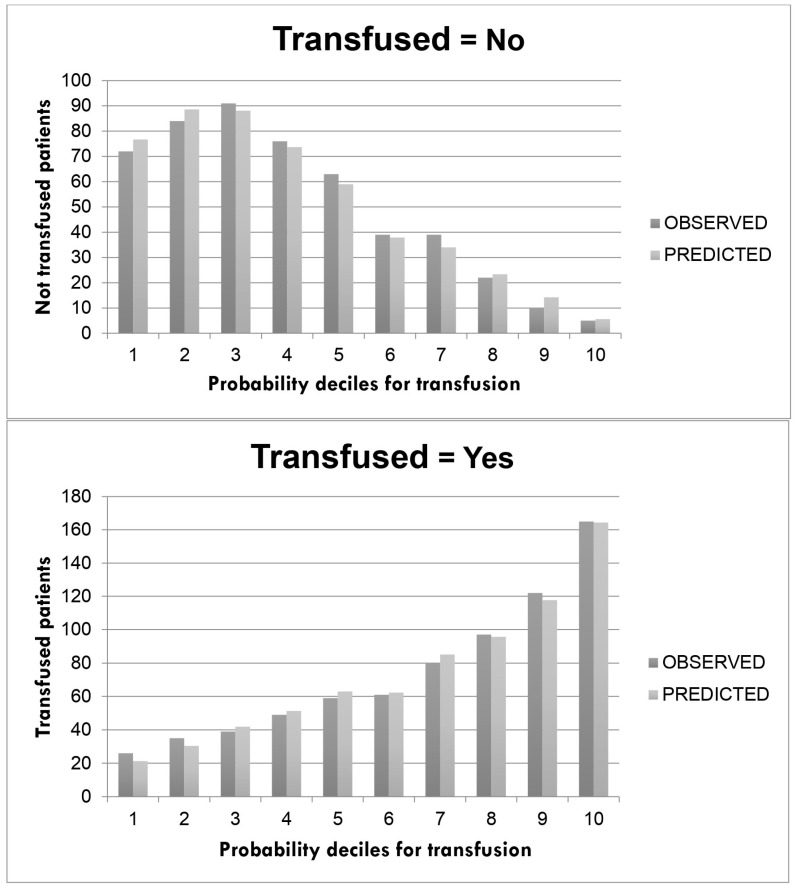
Calibration plots for transfusion prediction according to the proposed multivariable model.

After the univariate and multivariable analyses, only five factors in our model were included in the final model: age, sex, BMI, perioperative Hb and intervention. Table 4 shows the risk model for each factor. The decile of the probability of transfusion observed and predicted by the model is included in Figure 1. The final model shows an AUC for the prediction of transfusion risk with 80.9% accuracy ([CI 95% (78.5–83.3%)]; *p* < 0.001) (Figure 2).

## 4. Discussion

In our study, we developed a simple and parsimonious model of transfusion risk in cardiac surgery. Our resulting model comprises common variables collected for all patients, including sex, age, BMI, type of procedure, and preoperative Hb level. None of these variables, except preoperative Hb, requires additional tests. It is indeed true that before any cardiac surgery procedure, a preoperative Hb test is essential.

Incorporating numerous variables to “adjust” the data can pose a potential risk for the accuracy of the results. Therefore, statisticians typically recommend adopting a parsimonious approach when it comes to selecting independent variables. However, if a simple model can achieve the same level of accuracy in explaining a phenomenon as complex models, the principle of “parsimony” or “Occam’s razor” suggests that this simpler model should be favored, at least until a superior, more complex model emerges. The accuracy of classification in our model was 0.81 (95% CI 0.78; 0.83), which was higher than Klein’s external validation cutoff [8] of 0.76 (95% CI 0.75; 0.77). The TRACK model [11] has an AUC of 0.78, 95% CI (0.72; 0.74), while the TRUST model [10] has an AUC of 0.79, 95% CI (0.78; 0.80). These models require more information about the patient undergoing cardiac surgery, making them more complex for use in daily clinical practice. Furthermore, the latter model only calculates the probability of needing a transfusion in the intraoperative and postoperative period. We can observe that our model classifies the sample in an adequate way when compared to other models proposed in the literature and is also simpler, using fewer variables. Other models, such as the Goudie model [7], have an AUC of 0.77, 95% CI (0.77; 0.78), but it is not comparable to ours, as it is based on the risk of transfusion of any type of blood product.

In our study population, a total of 733 (59.4%) patients required transfusion, which was greater than the number of patients in the study by Klein et al. (43%) and in the TRUST model (51.5%), and lower than that in the TRACK model (98%) [8,10,11]. The mean patient age in our model was 63 years, while it was 67 years in the ACTA–PORT model and 62.5 years in the TRUST model [8,10]. However, our sample did not follow a normal distribution; we used the median age, which was 66 years. Mean preoperative hemoglobin levels were similar between our model (12.99 g/dL), the TRUST model (13.4 g/dL), and Klein’s work (13.2 g/dL) [8,10].

In our sample, 65.1% of the patients had HBP, which was consistent with the samples of Klein (67.8%) and Karkouti et al. (59%) [8,21]. Among the hypertensive patients, 56.7% of our population required transfusion compared to 68.6% of Klein’s sample [8].

Regarding DM, 31.4% of our sample had DM, and 24.8% of these patients required transfusion. In Klein’s sample, 22% had DM, and 23.8% of these patients required transfusion, while Karkouti reported DM in 27% of their population [8,21].

Combined surgery had a higher risk of transfusion in both Klein’s study and ours, consistent with Hardy et al.’s 1991 study [8,22]. In our sample, 69.4% of cases were isolated CABG or valve replacement surgeries, and 57.3% of those required transfusion. In Klein’s work, 73% were isolated CABG or valve replacement surgeries, and 39% required transfusion [8]. Combined surgery represented 14% of Klein’s total, and 65% of those patients required transfusion [8]. In our work, this surgery was performed in 9.6% of cases and 86.6% of these required transfusion. The multivariable model of Klein et al. showed that combined surgery had a 2.84-fold greater risk of transfusion than isolated coronary bypass surgery or valve replacement surgery, while our model showed a 1.38-fold greater risk than isolated surgery [8].

In both our study and Klein’s, patients with preoperative hemoglobin levels < 13 g/dL received approximately 50% of transfusions [8]. This suggests that optimizing preoperative hemoglobin levels through preoperative transfusions, iron supplements, the use of EPO, or other measures could be modify this risk factor to reduce the need for transfusions.

All models include the variable of age and sex, except for the Karkouti model [21]. It should be noted that the TRUST model and the Goudie model use some of the variables included in the calculation of EuroSCORE I, and Klein et al. uses it in the ACTA–PORT model [7,8,10].

Furthermore, it should be noted that the inclusion of the EuroSCORE I variable in a model can limit its use in populations where EuroSCORE I has not been validated and unique predictors of mortality in cardiac surgery may exist, such as Australia or China [23,24]. Another factor that almost all models use is preoperative hemoglobin or, failing that, hematocrit, as these are the most important variables when it comes to the risk of requiring a transfusion and where one can intervene to improve results.

We observed a link between cardiovascular risk factors (HBP, DM, DLP, sex, and age) and transfusion risk. The prevalence of diabetes in patients undergoing cardiac surgery is around 20% and is a risk factor for coronary artery disease associated with worse surgical outcomes [25]. Hypertension affects 80% of patients undergoing cardiac surgery and increases the risk of bleeding, myocardial ischemia, stroke and neurocognitive dysfunction [26].

This type of scoring system by the surgical team can be used to assess the likelihood of a transfusion requirement before a surgical procedure. This evaluation helps mitigate certain risk factors before surgery, leading to better planning of resources during the intervention. Furthermore, the use of other models, such as Papworth’s bleeding risk, permits preoperative categorization of patients based on their bleeding risk in the immediate postoperative period as low, medium or high, allowing for a more personalized approach to the treatment of patients [27].

The ability to optimize certain characteristics of the patient can help reduce the number of transfusions, thus avoiding various complications associated with transfusions such as infection, acute renal failure, increased risk of ischemia, acute pulmonary failure and the double risk of mortality at 5 years demonstrated by the use of transfusions [2,4,5,28]. Reducing transfusion frequency would allow costs to be reduced, allowing blood reserves to be made available to patients who need them more and minimizing expenditure on these scarce products.

Furthermore, it should be noted that in 2008, Koch et al. demonstrated that the duration of storage of red blood cells is associated with morbidity and mortality in cardiac surgery patients, and that transfusions with blood stored for more than 14 days are associated with a significant increase in hospital mortality [29]. However, recent studies did not find an association between storage time and a worse surgery outcome [30,31].

Some of our future research goals based on this work are to perform an analysis of all blood products used at different times during the perioperative period, to perform a cost-effectiveness analysis and to evaluate the losses of products that are discarded when the cold chain is broken at some point. These products should not be refrigerated again after 30 min and must be administered before 30 min have elapsed since removal from the refrigerator. In this way, we can minimize the losses of these precious products.

### Limitations of the Study

The main limitation is that we performed cross-validation with the ACTA–PORT model by attempting to replicate this model using the same variables; however, we were unable to achieve model duplication. Furthermore, this is a single-center study with a moderate sample size, and since the study is retrospective in nature, the data should be considered exploratory. Therefore, a new prospective study, conducted in several centers and with a larger sample size, would be necessary for validation.

Another limitation of this study is that it does not consider the administration of other blood products, the use of which may affect postoperative outcomes. Therefore, in the future, a study should be conducted that includes all blood products transfused during the perioperative period.

Certain cardiac transplant surgeries and emergency surgeries have been excluded from the study due to their high resource consumption, which could alter the results.

## 5. Conclusions

In conclusion, we propose a predictive model for the risk of red blood cell transfusion in cardiac surgery that has good discriminant capacity, is parsimonious, and is more efficient than other models developed for the same purpose. The following factors have been identified as hypothetical predictors of the need for blood transfusion in patients undergoing cardiac surgery: age, sex, body mass index, type of surgery, and preoperative hemoglobin, all of which are accessible and easy to use.

## Figures and Tables

**Table 1:** Description of the entire cohort.

	Total N = 1234
Age (years), median, (IQR)	66 (58.0; 73.0)
Sex male, *n* (%)	789 (63.9)
BMI, median, (IQR)	28.2 (25.3; 31.8)
BSA median, (IQR)	1.8 (1.7; 2.0)
Preoperative Hb (g/dL), median, (IQR)	13.0 (11.7; 14.2)
Serum creatinine (μmol/L), preoperative median, (IQR)	0.9 (0.8; 1.1)
EuroSCORE I median, (IQR)	4.4 (2.4; 7.0)
Current smoker	227 (18.4)
High blood pressure, *n* (%)	803 (65.1)
Diabetes mellitus, *n* (%)	387 (31.4)
Dyslipidemia, *n* (%)	629 (51.0)
Surgery, *n* (%)	
One procedure	857 (69.4)
Combined surgery	119 (9.6)
Other procedures	258 (20.9)
Patient transfused, *n* (%)	733 (59.4)
Median number of transfusions	1 (0; 2)
ACTA–PORT score median, (IQR)	15 (10; 18)
Median time of extracorporeal circulation (min), (IQR)	109 (85; 140)
Median time of ICU stay (days) (IQR)	3 (2; 4)
Median time of hospital stay median (days) (IQR)	14 (9; 24)
Mortality, *n* (%)	57 (4.6)

**Table 2:** Patient characteristics according to surgery procedures.

	One Procedure N = 857		Combined Procedure N = 119		Other Procedures N = 258		
	N (%)	CI 95.0%	N (%)	CI 95.0%	N (%)	CI 95.0%	*p*-Value
Age (years), median, (IQR)	66 (59; 73)	(66; 68)	69 (64; 75)	(67; 72)	60 (46; 69)	(56; 63)	<0.001
Sex male, *n* (%)	554 (64.6)	(61.4; 67.8)	74 (62.2)	(53.3; 70.5)	161 (62.4)	(56.4; 68.1)	0.738
BMI, median, (IQR)	28.2 (25.3; 27.9)	(27.9; 28.7)	28.2 (25.3; 31.9)	(27.5; 29.4)	28.3 (25; 31.9)	(27.6; 29.4)	0.904
BSA, median	1.8 (1.7; 2.0)	(1.8; 1.8)	1.8 (1.7; 2.0)	(1.8; 1.9)	1.9 (1.7; 2.0)	(1.8; 1.9)	0.143
Preoperative Hb (g/dL), median, (IQR)	13.0 (11.8; 14.2)	(12.9; 13.2)	12.2 (11; 13.5)	(11.9; 12.6)	13.5 (12; 14.6)	(13.3; 13.8)	<0.001
Serum creatinine (μmol/L), preoperative median, (IQR)	0.95 (0.8; 1.15)	(0.94; 0.97)	0.96 (0.82; 1.15)	(0.9; 1.04)	0.92 (0.79; 1.10)	(0.89; 0.97)	0.129
EuroSCORE I median, (IQR)	3.90 (2.27; 6.30)	(3.73; 4.09)	4.99 (3.29; 7.88)	(4.2; 5.5)	5.48 (3.29; 10.36)	(5.41; 6.35)	<0.001
Current smoker	165 (19.3)	(16.7; 22.0)	18 (15.1)	(9.6; 22.4)	44 (17.1)	(12.8; 22.0)	0.249
High blood pressure, *n* (%)	585 (68.3)	(65.1; 71.3)	85 (71.4)	(62.9; 79.0)	133 (51.6)	(45.4; 57.6)	<0.001
Diabetes mellitus, *n* (%)	304 (35.5)	(32.3; 38.7)	62 (52.1)	(43.2; 60.9)	21 (8.1)	(5.3; 11.9)	<0.001
Dyslipidemia, *n* (%)	472 (55.1)	(51.8; 58.4)	79 (66.4)	(57.6; 74.4)	78 (30.2)	(24.9; 36.0)	<0.001
Patient transfused, *n* (%)	491 (57.3)	(54; 60.6)	103 (86.6)	(79.6; 91.8)	139 (53.9)	(47.8; 59.9)	<0.001
Median number of transfusions	1 (0; 2)	(1; 2)	2 (1; 3)	(2; 3)	0 (0; 2)	.	<0.001
ACTA–PORT score median, (IQR)	15 (11; 18)	(15; 16)	19 (15; 23)	(18; 21)	12 (8; 16)	(11; 13)	<0.001
Median time of extracorporeal circulation (min), (IQR)	100 (81; 127)	(98; 103)	133.5 (113; 156)	(125; 140)	133.5 (94; 178)	(125; 145)	<0.001
Median time of ICU stay (days) (IQR)	3 (2; 4)	(3; 4)	4 (2; 5)	(4; 5)	3 (2; 5)	(3; 4)	0.002
Median time of hospital stay median (days) (IQR)	14 (9; 24)	(13; 15)	17 (12; 28)	(16; 22)	12 (9; 22)	(11; 15)	<0.001
Mortality *n* (%)	30 (3.5)	(2.4; 4.9)	12 (10.1)	(5.6; 16.4)	15 (5.8)	(3.43; 9.2)	0.004

**Table 3:** Patient characteristics according to transfusion.

	Total N = 1234	Not Transfused N = 501	Transfused N = 733	*p*-Value
Age (years), median, (IQR)	66 (58.0; 73.0)	63 (54; 71)	67 (61; 74)	<0.001
Sex male, *n* (%)	789 (63.9)	382(76.2)	407 (55)	<0.001
BMI, median, (IQR)	28.2 (25.3; 31.8)	29 (25.8; 32)	28.01 (25.0; 31.6)	0.013
BSA, median	1.8 (1.7; 2.0)	1.94 (1.80; 2.07)	1.83 (1.69; 1.99)	<0.001
Preoperative Hb (g/dL), median, (IQR)	13.0 (11.7; 14.2)	14.1 (13.2; 15)	12.2 (11; 13.3)	<0.001
Serum creatinine (μmol/L), preoperative median, (IQR)	0.9 (0.8; 1.1)	0.92 (0.79; 1.18)	0.97 (0.79; 1.18)	0.028
EuroSCORE I median, (IQR)	4.4 (2.4; 7.0)	3.51 (2.1; 5.5)	5.13 (3.13; 8.1)	<0.001
Current smoker	227 (18.4)	95 (19.0)	132 (18.0)	0.671
High blood pressure, *n* (%)	803 (65.1)	284 (56.7)	519 (70.8)	<0.001
Diabetes mellitus, *n* (%)	387 (31.4)	124 (24.8)	263 (35.9)	<0.001
Dyslipidemia, *n* (%)	629 (51.0)	223 (44.5)	406 (55.5)	<0.001
Surgery, *n* (%)				<0.001
One procedure	857 (69.4)	366 (73.1)	491 (67.0)	
Combined surgery	119 (9.6)	16 (3.2)	103 (14.1)	
Other procedures	258 (20.9)	119 (23.8)	139 (19.0)	
ACTA–PORT score, median, (IQR)	15 (10; 18)	11 (9; 14)	17 (13; 20)	<0.001
Median time of extracorporeal circulation (min), (IQR)	109 (85; 140)	100 (78; 128)	118 (89; 148)	<0.001
Median time of ICU stay (days), (IQR)	3 (2; 4)	2 (2; 4)	3 (2; 5)	<0.001
Median time of hospital stay median (days), (IQR)	14 (9; 24)	10 (9; 15)	19 (11; 28)	<0.001
Mortality, *n* (%)	57 (4.6)	0 (0)	57 (7.8)	<0.001

**Table 4:** Final model for predicting the need for perioperative blood transfusion in cardiac surgery.

	β	Standard Error	OR	CI 95%	*p* Value
Hb preoperative					
≥14 g/dL			1		<0.001
13–13.9 g/dL	0.74	0.17	2.11	1.50; 2.97	<0.001
12–12.9 g/dL	1.58	0.19	4.88	3.34; 7.10	<0.001
11–11.9 g/dL	2.38	0.25	10.89	6.69; 17.72	<0.001
<11 g/dL	3.94	0.43	51.41	21.97; 120.27	<0.001
Surgery					
One procedure			1		
Combined surgery	1.38	0.30	3.97	(2.19; 7.17)	<0.001
BMI					
BMI <30			1		
BMI ≥30	0.38	0.14	1.46	(1.10; 1.93)	<0.001
Sex					
Men			1		
Women	0.51	0.15	1.67	(1.24; 2.24)	0.001
Age					
<60			1		
≥60	0.31	0.15	1.37	(1.02; 1.83)	0.033
Constant	−1.46	0.18	0.23		<0.001

## Data Availability

The data presented in this study are available upon request from the corresponding author.

## References

[1] Raphael J., Mazer C.D., Wilkey A., Subramani S., Schroeder A., Abdalla M., Ferreira R., Roman P.E., Welsby I., Greilich P.E. (2020). Corrigendum to ‘Society of Cardiovascular Anesthesiologists (SCA) Clinical Practice Improvement (CPI) Advisory for Management of Perioperative Bleeding and Hemostasis in Cardiac Surgery Patients. J. Cardiothorac. Vasc. Anesth..

[2] Engoren M.C., Habib R.H., Zacharias A., Schwann T.A., Riordan C.J., Durham S.J. (2002). Effect of blood transfusion on long-term survival after cardiac operation. Ann. Thorac. Surg..

[3] Shaw R.E., Johnson C.K., Ferrari G., Brizzio M.E., Sayles K., Rioux N., Zapolanski A., Grau J.B. (2014). Blood transfusion in cardiac surgery does increase the risk of 5-year mortality: Results from a contemporary series of 1714 propensity-matched patients. Transfusion.

[4] Horvath K.A., Acker M.A., Chang H., Bagiella E., Smith P.K., Iribarne A., Kron I.L., Lackner P., Argenziano M., Ascheim D.D. (2013). Blood transfusion and infection after cardiac surgery. Ann. Thorac. Surg..

[5] Johnson D.J., Scott A.V., Barodka V.M., Park S., Wasey J.O., Ness P.M., Gniadek T., Frank S.M. (2016). Morbidity and Mortality after High-dose Transfusion. Anesthesiology.

[6] Al-Khabori M., Al-Riyami A.Z., Mukaddirov M., Al-Sabti H. (2014). Transfusion indication predictive score: A proposed risk stratification score for perioperative red blood cell transfusion in cardiac surgery. Vox Sang..

[7] Goudie R., Sterne J.A.C., Verheyden V., Bhabra M., Ranucci M., Murphy G.J. (2015). Risk scores to facilitate preoperative prediction of transfusion and large volume blood transfusion associated with adult cardiac surgery. Br. J. Anaesth..

[8] Klein A., Collier T., Yeates J., Miles L., Fletcher S., Evans C., Richards T. (2017). The ACTA PORT-score for predicting perioperative risk of blood transfusion for adult cardiac surgery. Br. J. Anaesth..

[9] Pajares A., Larrea L., Zarragoikoetexea I., Tur A., Vicente R., Argente P. (2018). Patient blood management in cardiac surgery: Results. Rev. Española Anestesiol. Reanim. (English Ed.).

[10] Alghamdi A.A., Davis A., Brister S., Corey P., Logan A. (2006). Development and validation of Transfusion Risk Understanding Scoring Tool (TRUST) to stratify cardiac surgery patients according to their blood transfusion needs. Transfusion.

[11] Ranucci M., Castelvecchio S., Frigiola A., Scolletta S., Giomarelli P., Biagioli B. (2009). Predicting transfusions in cardiac surgery: The easier, the better: The Transfusion Risk and Clinical Knowledge score. Vox Sang..

[12] Da Cunha C.B.C., Monteiro V.S., de Magalhães Ferraz D.L., Tchaick R.M., de Carvalho Júnior J.D., Silva I.T.C., Figueira F.A.M.D.S., Andrade L.B. (2023). Validation of Blood Transfusion Risk Scores (TRACK and TRUST) in a Cardiac Surgery Service in Brazil. Braz. J. Cardiovasc. Surg..

[13] Madhu Krishna N.R., Nagaraja P.S., Singh N.G., Nanjappa S.N., Kumar K.N., Prabhakar V., Manjunatha N. (2019). Evaluation of Risk Scores in Predicting Perioperative Blood Transfusions in Adult Cardiac Surgery. Ann. Card. Anaesth..

[14] Lemeshow S., Gauducheau E., Roques F., Nashef S.A.M., Michel P., Salamon R. (1999). European system for cardiac operative risk eval-uation (EuroSCORE). Eur. J. Cardio-Thoracic. Surg..

[15] Vlot E.A., Vernooij L.M., Loer S.A., van Dongen E.P., Noordzij P.G. (2022). External Validation of the ACTA-PORT Transfusion Risk Score in Older Cardiac Surgery Patients at Risk of Frailty. J. Cardiothorac. Vasc. Anesth..

[16] Leff J., Romano C.A., Gilbert S., Nair S. (2019). Validation Study of the Transfusion Risk and Clinical Knowledge (TRACK) Tool in Cardiac Surgery Patients: A Retrospective Analysis. J. Cardiothorac. Vasc. Anesth..

[17] Carpenter J., Bithell J. (2000). Bootstrap Confidence Intervals: When, Which, What? A Practical Guide for Medical Statisticians. Stat. Med..

[18] Kleinbaum D.G., Kleinbaum D.G. (2008). Applied Regression Analysis and Other Multivariable Methods.

[19] Bulus M. (2023). Pwrss: Statistical Power and Sample Size Calculation Tools. R Package Version 0.3.1. https://CRAN.R-project.org/package=pwrss.

[20] R Core Team (2020). R: A Language and Environment for Statistical Computing.

[21] Karkouti K., Yau T.M., Scott Beattie W., Callum J., Cheng D., Dupuis J.-Y., Kent B., Laflamme C., Légaré J.-F., Mazer D. (2006). Prediction of massive blood transfusion in cardiac surgery. Can. J. Anesth..

[22] Hardy J.-F., Perrault J., Tremblay N., Robitaille D., Blain R., Carrier M. (1991). The stratification of cardiac surgical procedures according to use of blood products: A retrospective analysis of 1480 cases. Can. J. Anaesth..

[23] Reid C., Billah B., Dinh D., Smith J., Skillington P., Yii M., Seevanayagam S., Mohajeri M., Shardey G. (2009). An Australian risk prediction model for 30-day mortality after isolated coronary artery bypass: The AusSCORE. J. Thorac. Cardiovasc. Surg..

[24] Zheng Z., Zhang L., Li X., Hu S. (2013). SinoSCORE: A logistically derived additive prediction model for post-coronary artery bypass grafting in-hospital mortality in a Chinese population. Front. Med. China.

[25] Moorthy V., Liu W., Chew S.T.H., Ti L.K. (2019). Impact of diabetes on outcomes of cardiac surgery in a multiethnic Southeast Asian population. Diabetes Vasc. Dis. Res..

[26] Aronson S., Dyke C.M., Stierer K.A., Levy J.H., Cheung A.T., Lumb P.D., Kereiakes D.J., Newman M.F. (2008). The ECLIPSE trials: Comparative studies of clevidipine to nitroglycerin, sodium nitroprusside, and nicardipine for acute hypertension treatment in cardiac surgery patients. Obstet. Anesth. Dig..

[27] Vuylsteke A., Pagel C., Gerrard C., Reddy B., Nashef S., Aldam P., Utley M. (2011). The Papworth Bleeding Risk Score: A stratification scheme for identifying cardiac surgery patients at risk of excessive early postoperative bleeding. Eur. J. Cardio-Thoracic Surg..

[28] Semple J.W., Rebetz J., Kapur R. (2019). Transfusion-associated circulatory overload and transfusion-related acute lung injury. Blood Am. Soc. Hematol..

[29] Koch C.G., Li L., Sessler D.I., Figueroa P., Hoeltge G.A., Mihaljevic T., Blackstone E.H. (2008). Duration of Red-Cell Storage and Complications after Cardiac Surgery. N. Engl. J. Med..

[30] Voorhuis F.T.R., Dieleman J.M., de Vooght K.M.K., van Dijk D., van Herwerden L.A., Peelen L.M., van Klei W.A. (2013). Storage time of red blood cell concentrates and adverse outcomes after cardiac surgery: A cohort study. Ann. Hematol..

[31] Xiao K., Zhao F., Liu Q., Jiang J., Chen Z., Gong W., Zheng Z., Le A. (2020). Effect of red blood cell storage duration on outcomes of isolated traumatic brain injury. Med. Sci. Monit..

